# Accuracy of Diagnosis of Human Granulocytic Anaplasmosis in China

**DOI:** 10.3201/eid2210.160161

**Published:** 2016-10

**Authors:** Gary P. Wormser

**Affiliations:** New York Medical College, Valhalla, New York, USA

**Keywords:** anaplasma, human granulocytic anaplasmosis, nosocomial infections, severe fever with thrombocytopenia syndrome, blood parasite, bacteria, China, parasites

## Abstract

In 2008, human granulocytic anaplasmosis (HGA) was reported from China. However, the clinical and laboratory findings, including reports of nosocomial transmission, were inconsistent with those reported for HGA in the United States. In 2012, it was demonstrated that the patients described in the 2008 report had all been infected with a newly discovered bunyavirus, severe fever with thrombocytopenia syndrome virus, which causes an illness with the same clinical features described for the patients in the 2008 report. This finding raises the question of HGA misdiagnosis in China and establishes the need for further studies to determine whether HGA occurs there.

## Abstract 2

Severe fever with thrombocytopenia syndrome may be misdiagnosed as human granulocytic anaplasmosis.

The first clinical report of human granulocytic anaplasmosis (HGA) in China was published in 2008 (*1*). This publication was also the first and only report claiming human-to-human transmission of *Anaplasma phagocytophilum* (or, to my knowledge, of any rickettsial agent in the absence of a blood transfusion or a needlestick) and is frequently cited in articles on tickborne infections (*2*,*3*).

To determine the accuracy of the report from China, I compared certain clinical and laboratory features of the 9 laboratory-confirmed cases of HGA in that outbreak (all secondary case-patients were claimed to have been infected by the index patient) (*1*) with 44 culture-confirmed cases of HGA reported from a study conducted in the United States (*4*) (Table 1). The 9 China cases differed from the 44 US cases in that the patients from China were significantly less likely to report headache but significantly more likely to have diarrhea, leukopenia, severe leukopenia (<3,000 leukocytes/mm^3^), and thrombocytopenia (including more severe thrombocytopenia of <100,000 platelets/mm^3^). As was pointed out in the editorial that accompanied the report of HGA in China (*5*), other noteworthy differences were observed between the HGA patients in China and those in the United States. One of these differences was relative bradycardia in all 9 patients in China (*1*), a finding never reported for HGA in the United States. Another difference was convalescent antibody titers against *A. phagocytophilum* of <1:256 for all 9 patients in China, with testing performed by using an IgG immunofluorescent antibody kit (Focus Diagnostics, Cypress, CA, USA) (*1*), whereas titers of >1:640 were documented for ≈95% of 44 culture-positive HGA patients in the United States (*4*). In addition, for none of the 9 China patients were morulae observed on blood smear, despite the fact that no patient had received a tetracycline antimicrobial drug, and all patients were said to be positive for *A. phagocytophilum* DNA according to PCR (*1*). In contrast, morulae were seen for 34 (77.3%) of the 44 culture-positive US HGA patients (*4*). Furthermore, in the China case series, sequencing of the *gro*EL gene product amplified by nested PCR indicated that the sequence was more similar to that of strains of A. *phagocytophilum* from the United States than from China*,* consistent with the occurrence of laboratory contamination. Nested PCR testing is prone to contamination, and the increased sensitivity afforded by this testing method is usually unnecessary when bacteria are numerous enough to be visualized by microscopy, as would be anticipated for patients with HGA (*6*).

**Table 1:** Frequency of certain clinical and laboratory features of HGA in China versus the United States*

Characteristic	China, no. (%) patients†	United States, no. (%) patients‡	p value§
Headache	2 (22)	36 (82)	0.0011
Diarrhea	7 (78)	6 (14)	0.0003
Leukopenia			
<4,500 cells/mm^3^	9 (100)	24 (55)	0.0097
<3,000 cells/mm^3^	8 (89)	11 (25)	0.0006
Thrombocytopenia			
<150,000 platelets/mm^3^	9 (100)	27 (61)	0.0442
<100,000 platelets/mm^3^	8 (89)	13 (30)	0.0015

*HGA, human granulocytic anaplasmosis. †Initial case series, n = 9. ‡Culture-confirmed case series, n = 44. §By Fisher exact test.

The reported nosocomial transmission of *A. phagocytophilum*, in conjunction with the atypical clinical and laboratory test results, already raised questions as to whether the diagnosis of HGA was correct when the article was published (*5*). A potential breakthrough in understanding what type of infection the index patient and the 9 secondary case-patients may have actually had occurred in April 2011, when Yu et al. identified a novel bunyavirus in parts of China that coincided geographically with the earlier report of nosocomial acquisition of HGA (*7*). On the basis of clinical data reported in the article about the initial discovery of this bunyavirus (*7*), plus other studies that followed (*8*–*10*), this tickborne bunyavirus was found to be responsible for a febrile illness associated with leukopenia, thrombocytopenia, and gastrointestinal manifestations. In addition, this virus has been repeatedly shown to be transmissible from person to person through mucocutaneous exposure to the blood of an infected patient (*11*–*17*). Furthermore, relative bradycardia in persons with this infection has been reported (*8*). The bunyavirus is called severe fever with thrombocytopenia syndrome virus (SFTSV).

When the 9 HGA cases described in the initial report from China (*1*) were compared with the 81 cases of SFTSV infection described in the initial publication about this infection (*7*), no significant differences were found for any of the parameters assessed (Table 2). This similarity is in marked contrast to the differences found when compared with the 44 culture-confirmed HGA cases from the United States (Table 1) (*4*). SFTSV infection, however, has many distinctive features when compared with HGA in the United States (Table 3).

**Table 2:** Frequency of certain clinical and laboratory features of HGA versus SFTSV infection in China*

Characteristic	HGA, no. (%) patients†	SFTSV, no. (%) patients‡	p value§
Headache	2 (22)	10 (12)	0.3434
Diarrhea	7 (78)	34 (42)	0.0737
Leukopenia, <4,500 cells/mm^3^	9 (100)	64/74 (86)	0.5910
Thrombocytopenia, <150,000 platelets/mm^3^	9 (100)	69/73 (95)	1.0000

*HGA, human granulocytic anaplasmosis; SFTSV, severe fever with thrombocytopenia syndrome virus. †Initial case series, n = 9. ‡Initial case series, n = 81. §By Fisher exact test.

**Table 3:** Prominent differences between HGA in the United States and SFTSV infection in Asia*

Clinical sign	More common with SFTSV infection	More common with HGA
Bleeding	Yes	No
Death	Yes	No
Gastrointestinal symptoms	Yes	No
Headache	No	Yes
Leukopenia and lower leukocyte counts	Yes	No
Lymphadenopathy	Yes	No
Person-to-person transmission†	Yes	No
Proteinuria	Yes	No
Relative bradycardia†	Yes	No
Thrombocytopenia and lower platelet counts	Yes	No

*HGA, human granulocytic anaplasmosis; SFTSV, severe fever with thrombocytopenia syndrome virus. Blank cells indicate not more common. †Never reported in the United States.

Thus, it is logical to ask if the 9 HGA case-patients plus the index patient originally reported in China (*1*) actually had SFTSV infection. Liu et al. raised this same question and in their article published in 2012 demonstrated convincingly (i.e., by reverse transcription PCR, serologic testing, or both) that the index patient and the 9 secondary case-patients were infected with SFTSV (*11*). The article did not report, however, the results of retesting of the patient specimens for *A. phagocytophilum* DNA. Thus, there are only 2 potential hypotheses: 1) that all of the patients in the original China case series reported to have had HGA actually had SFTSV infection and not HGA, or 2) that all of the patients were co-infected with SFTSV and *A. phagocytophilum* (*18*,*19*).

The former hypothesis seems much more plausible because of the inconsistency of the laboratory test results for HGA in China with what would have been expected for this infection in the United States, in conjunction with the unprecedented and unlikely nosocomial transmission of *A. phagocytophilum* (*18*). In addition, the latter potential conclusion seems less likely in that it would mean that all 9 secondary case-patients were simultaneously infected with both infectious agents, without even 1 person having only 1 of the 2 infections, which is highly improbable. Considering that 23%–45% of the contacts became infected, depending on the time frame and proximity of the exposure to the index patient (*1*), and assuming that neither of these infections would per se affect the likelihood of person-to-person transmission of the other infection, it would be very unlikely that all secondary case-patients would have been co-infected with both pathogens. Assuming a binomial distribution, the likelihood that this would have occurred by chance alone is <0.0008 (Paul Visintainer, pers. comm., 2016 Jul 8). It also seems implausible that the frequency of headache would have been so low among these 9 case-patients if they were actually co-infected with HGA (Table 1). Why would simultaneous infection with SFTSV reduce the frequency of headache in HGA-infected patients?

To establish the recently proposed hypothesis that the index patient was co-infected with *A. phagocytophilum* and SFTSV by a single tick bite (*19*), several key pieces of information are required. One is whether the *Haemaphysalis longicornis* ticks (the primary tick vector for SFTSV) (*9*) found in the specific area of the Anhui Province of China where the index patient resided are infected with pathogenic strains of *A. phagocytophilum* (*5*); if so, the frequency of co-infection with SFTSV should be determined. In addition, it would be essential to establish whether this species of tick is a competent vector for *A. phagocytophilum* (*18*,*19*).

Since 2008, additional case series of patients with HGA in China have been reported (*20*–*22*). What is the accuracy of these HGA diagnoses? The clinical and laboratory features reported are rather atypical in comparison with those reported for US patients, including in certain case series a higher frequency of gastrointestinal symptoms, regional lymphadenopathy, hepatosplenomegaly, relative bradycardia, facial edema, proteinuria, elevated cardiac enzyme levels, bleeding, and death. Headache and myalgia were reported significantly less commonly in the case series of HGA in China than in the United States, whereas leukopenia and severe thrombocytopenia were reported significantly more frequently (Table 4) (*4*,*20*–*22*). It is unlikely that the patients in these case series from China (*20*–*22*) were actually infected with the novel *Anaplasma* species provisionally named *A. capra* because cytopenia is infrequent among patients infected with *A. capra* (*23*,*24*).

**Table 4:** Selected clinical and laboratory variables of patients with an HGA diagnosis in China and the United States*

Variable	China, no. (%) patients†	United States, no. (%) patients‡	p value§
Number	165	44	
Headache	38 (23.0)	36 (81.8)	<0.0001
Myalgias	44 (26.7)	33 (75.0)	<0.0001
Diarrhea	63 (38.2)	6 (13.6)	0.002
Leukopenia¶	144/145 (99.3)	24 (54.5)	<0.0001
<100,000 platelets/mm^3^	151 (91.5)	13 (29.5)	<0.0001

*HGA, human granulocytic anaplasmosis. †3 case series of HGA published in 2011, 2013, and 2015 (*20**–**22*). ‡Case series of culture-confirmed HGA published in 2013 (*4*). §Fisher exact test. ¶<4,500, <3,600, <4,000 leukocytes/mm^3^ for references (*4*), (*20*), and (*22*), respectively.

If the cases in the original article about nosocomial transmission were misdiagnosed as HGA (*1*), several cautions should ideally be exercised regarding the diagnosis of HGA in China, as well as in other geographic areas where HGA has not been previously diagnosed. One is that reliance on the US Centers for Disease Control and Prevention case definition of HGA in the United States, intended for surveillance purposes (*25*), may not be sufficient to justify a diagnosis of HGA in China at this time. Validation and standardization of the PCR testing methods for *A. phagocytophilum* used in China should be a priority. Furthermore, serologic titers of <1:640 should not be considered indicative of HGA infection (*26*). Low positive titers are common in China (up to 20%) (*27*) and in the United States (*26*) and by no means establish current or prior infection with *A. phagocytophilum*. Locally cultivated strains of *A. phagocytophilum* might be a preferred source of antigens for serologic testing. In addition, given the numerous well-documented examples of clinically misdiagnosed HGA in China before recognition of SFTSV infection (*28*), individual patient assessments and all newly published case series of HGA should include testing for this virus as well as for other relevant infections included in the differential diagnosis. To date, testing for SFTSV has not been reported in many of the published case series of HGA in China (*20*–*22*). More emphasis should be placed on establishing the diagnosis of HGA by using the microbiological standard of culturing the organism (*4*), at least until other diagnostic modalities can be adequately validated. To my knowledge, a positive culture result has been obtained for only 4 reported cases of HGA in China (*20*).

In conclusion, HGA cases have been reported from China since 2008. The clinical and laboratory features, including the claim of nosocomial transmission, differ markedly from the overall features of this infection in the United States. In retrospect, some of the HGA case-patients in China seem to have been infected with the newly discovered bunyavirus, SFTSV. Thus, I recommend that further efforts be made to validate laboratory testing for HGA in China.
